# Wearable Wireless EMG Sensors for Monitoring Post-Error Neuromuscular Responses During a Sport-Specific Inhibitory Control Task

**DOI:** 10.3390/bios16070362

**Published:** 2026-07-01

**Authors:** Mauricio Barramuño-Medina, Pablo Valdés-Badilla, Pablo Aravena-Sagardia, Jordan Hernandez-Martínez, Edgar Vásquez-Carrasco, Tatiana Romero-Arias, Claudio Bascour-Sandoval, Germán Gálvez-García

**Affiliations:** 1 Kinesiology Program, Faculty of Health Sciences, Universidad Autónoma de Chile, Temuco 4780000, Chile; mauricio.barramuno@uautonoma.cl; 2 Department of Physical Activity Sciences, Faculty of Education Sciences, Universidad Católica del Maule, Talca 3460000, Chile; 3 Sport Trainer Program, Faculty of Life Sciences, Universidad Viña del Mar, Viña del Mar 2520000, Chile; 4 Physical Education Pedagogy, Faculty of Education, Universidad Autónoma de Chile, Temuco 4780000, Chile; pablo.aravena@uautonoma.cl; 5 Department of Physical Activity Sciences, Universidad de Los Lagos, Osorno 5290000, Chile; jordan.hernandez@ulagos.cl; 6 Department of Education, Faculty of Humanities, Universidad de la Serena, La Serena 1700000, Chile; 7 Occupational Therapy School, Faculty of Psychology, Universidad de Talca, Talca 3465548, Chile; edgard.vasquez@utalca.cl; 8 Centro de Investigación en Ciencias Cognitivas, Faculty of Psychology, Universidad de Talca, Talca 3465548, Chile; 9 VITALIS Longevity Center, Universidad de Talca, Talca 3465548, Chile; 10 Departamento de Psicología Evolutiva y de la Educación, Facultad de Psicología y Logopedia, Universidad de La Laguna, Tenerife, 38296 Islas Canarias, Spain; tromeroa@ull.edu.es; 11 Department of Rehabilitation Sciences, Faculty of Medicine, Universidad de La Frontera, Temuco 4780000, Chile; 12 Department of Experimental Psychology, Psychobiology and Behavioral Sciences Methodology, Universidad de Salamanca, 37005 Salamanca, Spain; germangalvezgarcia@usal.es

**Keywords:** motor control, electromyography, reaction time, performance monitoring, biomechanical phenomena

## Abstract

Post-error slowing (PES) is commonly considered a behavioral marker of post-error adaptation. However, adaptive processes may also emerge through subtle modifications of motor preparation, particularly in combat sports such as taekwondo (TKD), where maintaining rapid motor execution is essential. This study examined post-error neuromuscular adjustments during a TKD-specific kicking task by comparing standard Go and post-error Go trials for changes in muscle onset latency, peak electromyographic amplitude, and co-contraction indices. Twenty-eight TKD athletes (14 novice and 14 advanced) performed a sport-specific Go/No-Go task while wearable wireless surface electromyography sensors recorded lower-limb neuromuscular activity from eight lower-limb muscles. Muscle onset latency, peak electromyographic amplitude, co-contraction indices, and reaction time were analyzed using linear mixed-effects models. Post-error Go trials showed significant alterations in muscle onset latency in posterior lower-limb muscles involved in propulsion and movement preparation (semitendinosus, biceps femoris, lateral gastrocnemius, and soleus), with muscle activation occurring closer to the foot take-off. No significant differences were observed in reaction time, peak electromyographic amplitude, or co-contraction indices, and expertise and age did not modulate these effects. These findings suggest that error-related motor adjustments may be expressed through changes in muscle activation timing rather than overt behavioral slowing.

## 1. Introduction

Adaptive behavior following errors is especially important in combat sports such as taekwondo (TKD), where performance relies on rapid perception and efficient processing of environmental cues under high temporal pressure and uncertainty [1,2]. During a bout, opponents frequently employ deceptive movements and feints to elicit incorrect responses and disrupt motor decision-making [3,4]. Consequently, errors may create brief windows of vulnerability in which athletes become more susceptible to counterattacks [2]. From this perspective, the ability to detect errors and rapidly adapt subsequent motor behavior represents a critical component of performance in TKD [4]. Importantly, these adaptive processes emerge within a continuous perception–action coupling framework, in which movement regulation is dynamically shaped by ongoing interactions between the performer and the environment [5].

From a cognitive–motor perspective, errors engage mechanisms of behavioral control and adjustment that modulate subsequent motor responses [6]. One of the most extensively studied manifestations of these adjustments is post-error slowing (PES), typically defined as an increase in reaction time following an error [7,8]. PES has traditionally been interpreted as an adaptive process reflecting increased response caution, allowing individuals to optimize performance by shifting along the speed–accuracy trade-off continuum [9]. However, accumulating evidence [10,11] indicates that post-error behavioral adjustments are not consistently expressed as slower responses. Although PES is frequently observed, its magnitude and even its presence appear to depend on task demands, motivational context, and strategic factors [6,11]. More recent evidence [10] further suggests that behavioral slowing is not necessarily associated with improved performance, indicating that post-error adaptation reflects a more complex set of processes than a uniform increase in caution.

Importantly, the absence of behavioral slowing does not necessarily imply the absence of post-error adaptation [10]. Behavioral measures such as reaction time provide only the final observable outcome of motor performance and may therefore fail to capture adjustments occurring during earlier stages of motor preparation [12]. More specifically, reaction time reflects the final behavioral output of performance, whereas muscle onset latency may provide access to the preparatory organization of motor output preceding movement execution [13,14]. In fast, sport-specific actions, maintaining response speed may be essential for successful performance, meaning that adaptive regulation may emerge through subtle modifications of motor preparation rather than overt slowing [10,15]. In combination, these findings suggest that post-error adaptation should not be conceptualized exclusively at the behavioral level. Rather, cognitive control processes may reorganize motor preparation and the muscle onset latency following errors. This motor implementation of cognitive control is supported by neurophysiological mechanisms, including corticospinal modulation and preparatory motor regulation, during action selection and motor adjustment [16,17]. Preparatory inhibitory processes appear to broadly modulate the motor system during movement preparation [17]. Such modulation may help reduce background motor noise and optimize signal processing prior to movement initiation [18]. In this sense, performance monitoring may act on an already-regulated preparatory motor system, modifying the organization of subsequent actions following an error. Performance-monitoring signals may transiently influence preparatory motor processes, contributing to adaptive motor recalibration following errors [6]. Such preparatory adjustments may involve selective modulation of motor readiness and preparatory circuits, facilitating the efficient organization of upcoming actions [12,14]. Within this framework, post-error adaptation may not necessarily involve gross suppression of motor output, but rather fine modulation of movement dynamics and preparatory timing [13]. Consequently, adjustments following errors may emerge as alterations in the muscle onset latency, even when overt behavioral performance remains relatively stable. Such adaptations may preferentially affect the muscle onset latency rather than the global magnitude of muscle activation, potentially reflecting optimization of preparatory motor regulation prior to movement initiation [12,13].

Surface electromyography (EMG) provides a valuable approach for examining these preparatory motor processes [19]. Unlike behavioral measures, EMG allows the characterization of muscle recruitment patterns underlying movement execution, including both the magnitude of muscle activation and muscle onset latency [19]. In particular, muscle onset latency represents a sensitive index of preparatory motor timing preceding overt movement execution [20]. From this perspective, post-error adjustments may manifest as adaptive motor recalibration through temporal reorganization of muscle recruitment timing, rather than as changes in overall activation magnitude. Thus, the muscle onset latency may represent a sensitive marker of cognitive–motor regulation following errors.

Despite the relevance of these mechanisms, the neuromuscular correlates of post-error adaptation remain poorly understood, particularly in ecologically valid sport-specific contexts. Most previous studies examining post-error processes have relied on simplified laboratory paradigms involving manual button-press responses, which provide limited insight into the complex neuromuscular coordination required during real-world motor actions [8,10,11]. Consequently, evidence regarding how errors influence the temporal organization of muscle activation during complex sport-specific movements remains limited. The present study addresses this gap by examining post-error neuromuscular responses during a taekwondo-specific kicking task using wearable surface electromyography, providing novel information on muscle activation timing, activation magnitude, and intermuscular coordination following errors in a combat-sport context. Combat sports such as TKD constitute an especially valuable model for studying these mechanisms because successful performance depends on rapid perception–action coupling, precise lower-limb coordination, and continuous online recalibration of motor preparation under dynamic conditions [5,21,22].

Accordingly, the present study examined post-error neuromuscular adjustments during a TKD-specific kicking task. Specifically, we compared standard Go trials and post-error Go trials to determine whether errors were associated with changes in muscle onset latency, peak EMG amplitude, and co-contraction indices. Additionally, given previous evidence [21,22,23] that TKD expertise is associated with more refined neuromuscular organization during kicking actions, we explored whether post-error adjustments were modulated by expertise and age. Based on the notion that post-error adaptation may emerge through subtle modifications in motor preparation processes. Consistent with previous evidence suggesting that preparatory motor regulation and corticospinal modulation may reorganize movement preparation without necessarily producing overt behavioral slowing [6,14,17], it was hypothesized that post-error Go trials would primarily exhibit alterations in the timing of muscle recruitment, particularly muscle onset latency, rather than changes in reaction time, peak EMG amplitude, or co-contraction indices. Additionally, given prior evidence that expertise in TKD is associated with more refined neuromuscular organization and movement preparation strategies [21,22], an exploratory hypothesis was formulated proposing that expertise and age might modulate the magnitude of post-error neuromuscular recalibration.

## 2. Materials and Methods

### 2.1. Study Design

**Figure 1:**
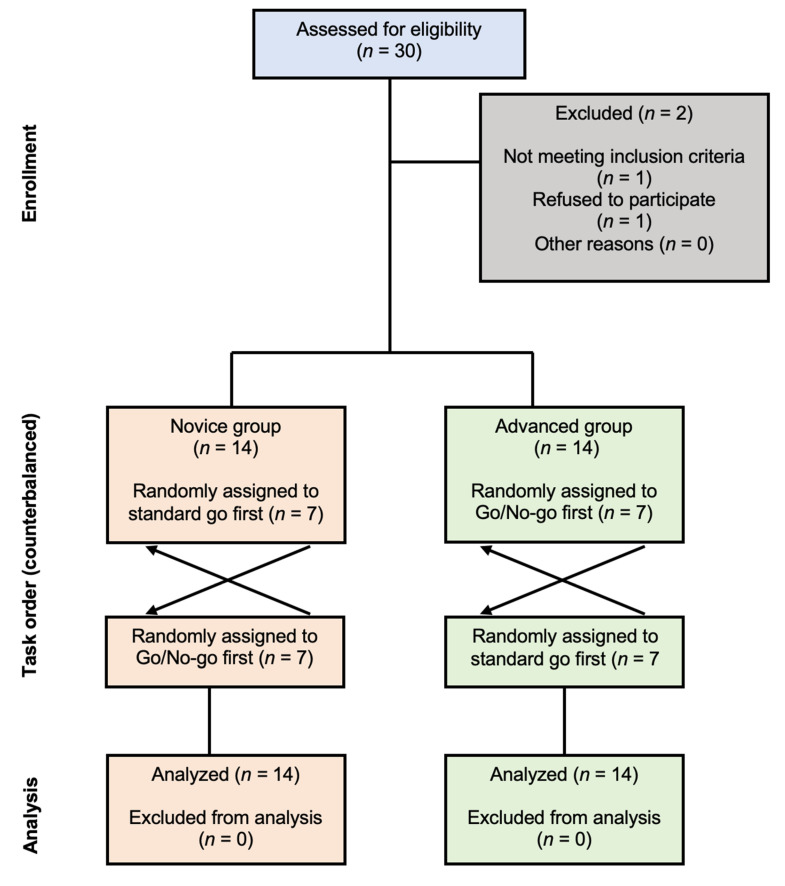
Study flowchart.

This study employed a cross-sectional repeated-measures design using a within-subject trial-by-trial approach to examine post-error neuromuscular adjustments during a TKD-specific motor task. Standard Go trials and post-error Go trials were compared within participants to assess immediate changes in motor performance following errors. The experimental design included multiple trials per participant, enabling repeated observations and improving the reliability of the estimates. Task order was randomized across participants in a counterbalanced manner to control for potential sequence effects (Figure 1). This approach enabled the characterization of within-subject variability in neuromuscular responses across conditions. The study was conducted and reported in accordance with the STROBE guidelines [24].

### 2.2. Participants

A total of 28 TKD athletes (19 females and 9 males) volunteered to participate in this study. Participants were allocated into two expertise groups: novice (*n* = 14) and advanced (*n* = 14). The novice group had a mean age of 13.8 ± 2.2 years, whereas the advanced group had a mean age of 19.0 ± 5.1 years. Recruitment was conducted through competitive TKD training programs, and all athletes were actively engaged in regular training during the data collection period. Expertise classification was based on both technical rank and training background. Athletes with a blue belt or higher and at least 3 years of systematic practice were assigned to the advanced group, whereas participants below this threshold and with less training experience were assigned to the novice group. Eligibility criteria included at least 1 year of TKD practice, participation in at least 2 weekly training sessions, active affiliation with the national federation (FEDENAT), and normal or corrected-to-normal vision. Participants were excluded if they presented musculoskeletal injuries at the time of testing, neurological or motor impairments, use of medication affecting neuromotor performance, or recent stimulant intake prior to assessment. The target sample size was informed by previous studies reporting expertise-related differences in inhibitory control among TKD athletes, as well as by methodological recommendations for repeated-measures research. Specifically, prior evidence has shown moderate-to-large effects associated with expertise level in inhibitory tasks [4]. Furthermore, repeated-measures designs using mixed or within-subject approaches can achieve stable parameter estimation with approximately 12–15 participants per group when an adequate number of observations per condition is obtained. Because no directly comparable study was available to estimate the expected effect of post-error neuromuscular adaptations during TKD kicking, no formal a priori sample size calculation was performed. Following data collection, a simulation-based post hoc power analysis was conducted in R (simr package) using the fitted linear mixed-effects models employed for the primary outcomes. Power was estimated from 1000 Monte Carlo simulations of the TrialType effect while maintaining the observed hierarchical structure of the data, including repeated observations nested within participants and participant-specific random intercepts. This procedure provided an assessment of statistical sensitivity that was consistent with the modeling approach used in the study. The estimated power was 90.0% for semitendinosus (ST), 93.0% for biceps femoris (BF), 80.8% for lateral gastrocnemius (LG), and 91.8% for soleus (SO), indicating sufficient sensitivity for the primary onset-latency outcomes. To strengthen within-subject reliability, the present protocol incorporated multiple repetitions across experimental conditions.

All participants, or their legal guardians when applicable, signed written informed consent before participation. The protocol received approval from the Research Ethics Committee of Universidad Autónoma de Chile (2 June 2025; N° CEC 12-25) and was conducted in accordance with the ethical principles outlined in the Declaration of Helsinki.

### 2.3. Experimental Procedure

Assessments were conducted individually between June 2025 and January 2026 at the Movement Analysis Laboratory of Universidad Autónoma de Chile. After written informed consent was obtained, demographic and expertise-related data were collected. Prior to testing, participants completed a predefined warm-up routine consisting of light aerobic activity, dynamic mobility exercises, and TKD-specific movements to ensure neuromuscular readiness and minimize injury risk. A familiarization phase comprising 20 practice trials was then performed to ensure that participants fully understood the stimulus–response rules and movement requirements of the task. Instructions emphasized rapid, accurate responses, highlighting both the need for fast execution during Go trials and for complete movement suppression during No-Go trials.

**Figure 2:**
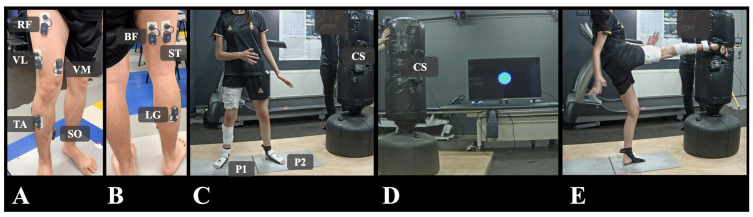
Experimental setup, sensor placement, and task execution. (**A**) Anterior view and (**B**) posterior view of lower-limb surface electromyography sensor placement. RF: rectus femoris; VL: vastus lateralis; VM: vastus medialis; TA: tibialis anterior; SO: soleus; BF: biceps femoris; ST: semitendinosus; LG: lateral gastrocnemius. (**C**) Standardized starting position adopted before stimulus presentation, showing the placement of each foot on the force platforms (P1 and P2) and the instrumented kicking target equipped with a contact sensor (CS). (**D**) Experimental environment illustrating the spatial arrangement of the participant, contact-sensing target (CS), and visual stimulus monitor. (**E**) Execution of the roundhouse kick toward the instrumented target. Participants wore taekwondo instep protectors throughout testing.

For the experimental task, athletes adopted a standardized TKD guard position with one foot placed on each force platform. All trials were performed barefoot while wearing standard TKD instep protectors. The experimental setup, including EMG sensor placement, force platforms, visual stimulus monitor, instrumented target, and task execution, is illustrated in Figure 2. In Go trials, participants executed a roundhouse kick with the dominant leg toward an instrumented target equipped with a contact sensor, whereas in No-Go trials, they were required to inhibit the kicking movement and maintain the initial stance. For trial-by-trial analyses, Go trials were classified according to the outcome of the immediately preceding trial. A commission error was defined as any instance in which the athlete initiated the kicking movement by lifting the foot from the force platform during a No-Go trial. Standard Go trials corresponded to correct Go trials preceded by another correct trial, whereas post-error Go trials corresponded to correct Go trials immediately following a commission error during a No-Go trial. This classification enabled the examination of immediate post-error neuromuscular adjustments in subsequent motor execution. The field-based Go/No-Go task was designed to preserve the core structure of standard computerized paradigms while incorporating sport-specific motor demands. The task was programmed using OpenSesame (OpenSesame project, Amsterdam, The Netherlands) [25]. Stimuli were presented on a 42″ monitor located 205 cm in front of the participant, ensuring a standardized visual angle across trials. Colored circles (blue or yellow) were presented pseudo-randomly at the center of the screen, and the stimulus–response mapping was counterbalanced across participants. Kicks were directed toward a padded target with an embedded contact sensor, individually adjusted to a body height between the sternum and chin level in accordance with TKD practice standards. The task consisted of five blocks of 20 trials each (16 Go and 4 No-Go trials; No-Go probability = 0.20). A variable intertrial interval ranging from 1500 to 2000 ms was used to reduce anticipatory responses and allow sufficient recovery between actions. Each block lasted approximately 1 min, with a 1 min rest period between blocks to minimize fatigue accumulation. The kicking task enabled the simultaneous acquisition of behavioral, kinetic, and EMG data. Force platforms were synchronized with the EMG acquisition system, allowing temporally aligned recordings. Movement initiation (foot take-off) was identified from the vertical ground reaction force signal. Behavioral performance was quantified using RT, defined as the time elapsed from stimulus presentation to target contact during correct Go trials.

### 2.4. Data Acquisition

EMG signals were obtained from eight lower-limb muscles using a wireless acquisition system (BTS Bioengineering, Milan, Italy) with a sampling rate of 1000 Hz. In parallel, ground reaction forces were acquired at 500 Hz, with both systems temporally synchronized during recording. Data processing was performed offline using custom MATLAB R2025a scripts (MathWorks, Natick, MA, USA). Prior to electrode placement, the skin was prepared by shaving and cleaning the area with alcohol to minimize impedance. Bipolar Ag/AgCl surface electrodes were positioned on the dominant leg in accordance with SENIAM recommendations [26]. Electrode locations were defined as follows: the biceps femoris (BF) was placed at the midpoint between the ischial tuberosity and the lateral femoral epicondyle; the semitendinosus (ST) at the midpoint between the ischial tuberosity and the medial tibial epicondyle; the rectus femoris (RF) at the midpoint between the anterior superior iliac spine and the superior border of the patella; the vastus medialis (VM) approximately 4 cm superior and medial to the patella; and the vastus lateralis (VL) at two-thirds of the distance between the ASIS and the lateral border of the patella. For the lower leg muscles, the gastrocnemius lateralis (LG) was positioned at one-third of the distance between the fibular head and the Achilles tendon on the lateral aspect of the muscle belly; the soleus (SO) at the midpoint between these landmarks on the medial side; and the tibialis anterior (TA) at one-third of the distance between the tibial tuberosity and the medial malleolus, positioned lateral to the tibial crest. For EMG normalization, maximal voluntary contractions (MVC) were obtained for each monitored muscle using standardized testing positions from SENIAM recommendations [26]. Participants were instructed to gradually increase force and then maintain a maximal isometric contraction against manual resistance. Three MVC trials were performed for each muscle, and the highest value obtained across trials was used as the normalization reference. For the RF, VM, and VL, MVC were obtained during maximal resisted knee extension in a seated position with the knee flexed to approximately 70°. The BF and ST were assessed during maximal resisted knee flexion in the prone position with the knee flexed to 60°, using slight external and internal tibial rotation to emphasize BF and ST activation, respectively. TA was obtained during resisted ankle dorsiflexion combined with inversion. SO was assessed during resisted plantarflexion with the knee flexed to approximately 90° to reduce gastrocnemius contribution, whereas LG was obtained during maximal resisted plantarflexion in a standing position. EMG amplitudes recorded during the experimental task were subsequently expressed as a percentage of MVC (%MVC).

### 2.5. Signal Processing

**Figure 3:**
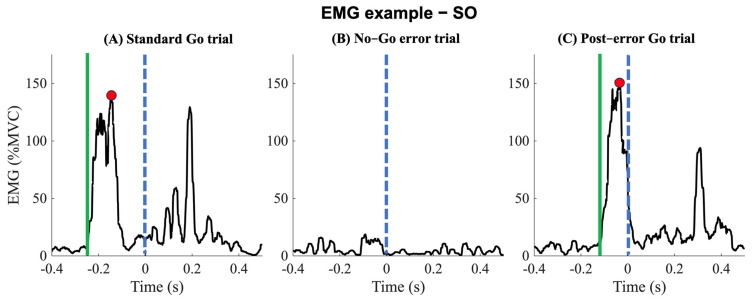
Representative electromyographic (EMG) signals for a selected muscle across three trial conditions: (**A**) Standard Go trial, (**B**) No-Go error trial, and (**C**) Post-error Go trial. Signals are expressed as a percentage of maximum voluntary contraction (%MVC) and aligned relative to the foot take-off. The blue dashed vertical line indicates the instant of foot take-off from the force platform (0 s). For the Go trials, muscle onset is identified by the green vertical line, and peak EMG amplitude within the activation burst is indicated by the red circle. Negative time values represent muscle activity occurring before foot take-off. SO: soleus.

Signal processing included noise reduction, feature extraction, and normalization procedures. EMG signals were band-pass filtered (30–450 Hz) using a zero-phase Butterworth filter to reduce movement artifacts associated with high-intensity dynamic actions [27]. A smoothed linear envelope was then obtained via root-mean-square (RMS) computation using a 20-sample moving window. To allow between-subject comparisons, EMG amplitudes were normalized to maximal voluntary contraction values and expressed as %MVC. A semi-automated algorithm was used to identify key temporal events within each trial (Figure 3). Foot take-off was initially detected from force platform data using a threshold-based approach applied to the first derivative of the force signal. When automatic detection was not sufficient, manual selection was performed to ensure accurate identification of the event. Muscle onset latency was determined using a semi-automated procedure that combined algorithm-based segmentation with visual inspection. Muscle onset latency was determined using a semi-automated threshold-based procedure. Raw EMG signals were band-pass filtered, rectified, and smoothed using an RMS moving window. Baseline activity was calculated from a pre-movement reference period, and the onset threshold was defined as the baseline mean plus three standard deviations. Muscle activation onset was identified as the first point at which the processed EMG signal exceeded the threshold and remained above it for a minimum duration of 25 ms. All automatically detected onsets were subsequently visually inspected by an experienced evaluator. Visual intervention was only performed when the algorithm failed to correctly identify the beginning of the primary activation burst (e.g., due to signal artifacts, secondary bursts, or atypical signal morphology). In such cases, the evaluator identified the appropriate activation burst, and the onset was subsequently re-established using the same threshold-based criterion. Accordingly, onset determination was primarily algorithm-based but included limited manual supervision when required. Peak EMG amplitude was computed within the same activation burst, defined as the maximum EMG value occurring within a 200 ms window following the identified onset. This approach ensured that peak values corresponded to the same functional phase of muscle activation across trials. Co-contraction indices (CCI) were calculated for selected antagonist muscle pairs over a time window defined from the earliest onset to the latest peak within each pair. The CCI was computed as the ratio of overlapping activation (minimum EMG between muscles) to total activation (sum of both EMG signals), providing an index of intermuscular coordination and joint stabilization [28]. To assess the reproducibility of the semi-automated onset-detection procedure, intra-rater and inter-rater reliability analyses were performed on a randomly selected subset of trials (~20% of the dataset). Both evaluators were blinded to TrialType and the original onset values. Reliability was quantified using intraclass correlation coefficients (ICC) with 95% confidence intervals and the standard error of measurement. The procedure demonstrated excellent reproducibility, with ICC values ranging from 0.998 to 1.000 and SEM values ranging from 0.65 to 1.68 ms.

### 2.6. Outcome Measures

The primary outcome was muscle onset latency (ms), defined as the timing of EMG activation relative to the foot take-off, reflecting preparatory motor organization. Secondary outcomes included RT, peak EMG amplitude (%MVC), and CCI. RT was defined as the time elapsed from stimulus presentation to target contact, as detected by the contact sensor. Peak EMG amplitude was defined as the maximum EMG value within a 200 ms window following muscle onset. CCIs were calculated for selected antagonist muscle pairs as the ratio between overlapping activation and total activation, providing an index of intermuscular coordination. Reaction time was defined as the interval between stimulus presentation and target contact, thereby reflecting both response initiation and movement execution components of the kicking task.

### 2.7. Statistical Analysis

Statistical analysis was conducted in R (version 4.5.1; R Foundation for Statistical Computing, Vienna, Austria) using RStudio (Posit Software, PBC, Boston, MA, USA). Linear mixed-effects models (LMMs) were fitted using the lme4 package [29]. Descriptive statistics are presented as mean (standard deviation), as appropriate. To examine differences between conditions, LMMs were used for all continuous outcomes, including muscle onset latency, peak electromyographic (EMG) amplitude, and co-contraction indices. In all models, Subject was included as a random intercept to account for repeated measures. All analyses were conducted at the trial level rather than on participant-level averages. Because the number of post-error Go trials depended on the occurrence of commission errors, the number of observations varied across participants. Linear mixed-effects models were selected because they appropriately accommodate unbalanced repeated-measures data and unequal numbers of observations per participant. Models including participant-specific random slopes for TrialType produced singular fits. Therefore, only random-intercept models were retained for the primary analyses. Competing models were specified to evaluate the effect of TrialType (Standard Go trials vs. post-error Go trials), both as a single predictor and in combination with covariates, including expertise and age (mean-centered). In addition, interaction terms (TrialType × Expertise and TrialType × Age) were examined. All models were fitted using maximum-likelihood estimation to enable model comparison. Model selection was performed using the Akaike Information Criterion corrected for small samples (AICc). The model with the lowest AICc was retained, with preference for parsimony when ΔAICc < 2. Model fit was further evaluated using marginal and conditional R^2^, calculated as described by Nakagawa. Model assumptions were assessed through visual inspection of residual-versus-fitted plots and normal Q-Q plots. Model convergence was verified for all fitted models, and no substantial deviations from normality or homoscedasticity were observed. Fixed effects were extracted from each model, and the effect of TrialType was systematically examined across model specifications. To control for multiple comparisons across variables, *p*-values were adjusted using the Benjamini–Hochberg false discovery rate (FDR) procedure. Statistical significance was set at *p* < 0.05 after correction.

## 3. Results

A total of 28 TKD athletes were included in the study and classified into two groups according to their level of expertise: novice (*n* = 14) and advanced (*n* = 14). The TKD novice group had a mean age of 13.8 ± 2.2 years, a body mass of 48.6 ± 8.8 kg, and a body height of 153.2 ± 10.2 cm. The TKD advanced group had a mean age of 19.0 ± 5.1 years, a body mass of 58.1 ± 12.6 kg, and a body height of 162.8 ± 8.6 cm. Sex distribution was similar between groups, with 10 females and 4 males in the TKD novice group and 9 females and 5 males in the TKD advanced group. The groups differed in training background. The TKD novice group reported 2.1 ± 0.9 years of experience and had participated in 6.2 ± 4.5 tournaments, whereas the TKD advanced group reported 8.7 ± 4.2 years of experience and 43.7 ± 19.8 tournaments. Each participant completed 100 trials (80 Go and 20 No-Go trials). Because post-error Go trials were contingent upon the occurrence of commission errors during No-Go trials, the number of post-error observations varied across participants. Participants contributed an average of 5.71 ± 2.95 post-error Go trials. Specifically, the novice group contributed 5.93 ± 2.87 trials, whereas the advanced group contributed 5.50 ± 3.03 trials. Participants contributed between 2 and 11 post-error Go trials, and four participants contributed fewer than four post-error observations.

### 3.1. Model Comparison

Across onset variables, the Trial-only model consistently provided the best fit, with AICc values ranging from approximately 571 to 669 and Akaike weights between 0.33 and 0.46. Marginal R^2^ values ranged from approximately 0.02 to 0.16, whereas conditional R^2^ values ranged from 0.22 to 0.49. A similar pattern was observed for peak EMG variables (AICc ≈ 500–618; R^2^m ≈ 0.00–0.03) and co-contraction indices (AICc ≈ −185 to −135; R^2^m ≈ 0.00–0.04; R^2^c ≈ 0.50–0.80).

### 3.2. Reaction Time

No significant effect of TrialType on reaction time was observed (β = 10.39 ms, SE = 18.20, t = 0.57, *p* = 0.573). Estimated marginal means indicated slightly longer reaction times in the post-error condition (797 ms) compared to standard Go trials (787 ms), although this difference was not statistically significant (*p* = 0.579) and was associated with a trivial effect size (d = 0.15).

### 3.3. Muscle Onset Latency

**Figure 4:**
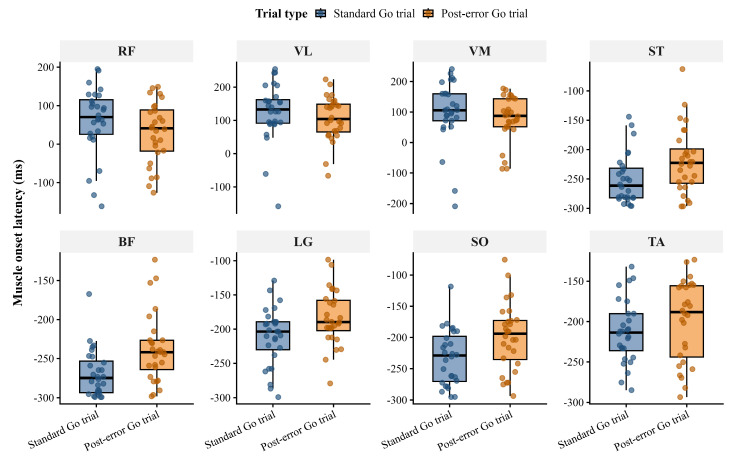
Muscle onset latency (ms) for each muscle across Standard Go trials and post-error Go trials. Boxplots represent the median and interquartile range, with individual data points overlaid. Negative values indicate activation occurring prior to the reference event. RF, rectus femoris; VL, vastus lateralis; VM, vastus medialis; ST, semitendinosus; BF, biceps femoris; LG, lateral gastrocnemius; SO, soleus; TA, tibialis anterior.

A significant main effect of TrialType was observed for ST, BF, LG and SO. No significant effects were observed for RF, VL, VM, or TA (all pFDR > 0.05). Detailed results are presented in Table 1 and illustrated in Figure 4.

### 3.4. Peak EMG

**Figure 5:**
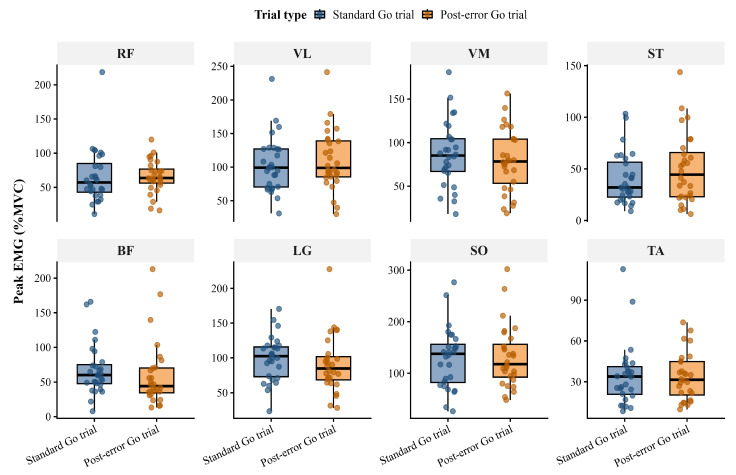
Peak electromyographic (EMG) amplitude (%MVC) for each muscle during Standard Go trials and post-error Go trials. Boxplots represent the median and interquartile range, with individual data points overlaid. RF, rectus femoris; VL, vastus lateralis; VM, vastus medialis; ST, semitendinosus; BF, biceps femoris; LG, lateral gastrocnemius; SO, soleus; TA, tibialis anterior.

A significant effect of TrialType was observed for ST peak EMG amplitude after FDR correction (pFDR = 0.049), whereas no significant effects were observed for the remaining muscles (all pFDR > 0.05). Specifically, ST peak EMG amplitude was higher during post-error Go trials compared with Standard Go trials. Detailed results are presented in Table 1 and illustrated in Figure 5.

### 3.5. Co-Contraction Indices

**Figure 6:**
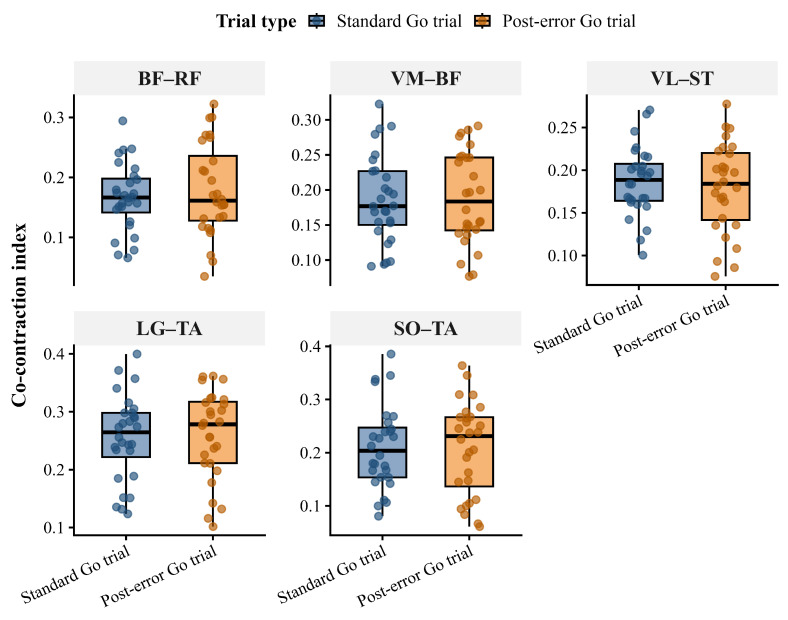
Co-contraction indices (CCI) for selected muscle pairs during Standard Go trials and post-error Go trials. Boxplots represent the median and interquartile range, with individual data points overlaid. The analyzed muscle pairs were BF–RF (biceps femoris–rectus femoris), VM–BF (vastus medialis–biceps femoris), VL–ST (vastus lateralis–semitendinosus), LG–TA (lateral gastrocnemius–tibialis anterior), and SO–TA (soleus–tibialis anterior).

No significant effects of TrialType were observed for co-contraction indices across all muscle pairs after FDR correction (all pFDR > 0.05). Detailed results are presented in Table 1 and illustrated in Figure 6.

## 4. Discussion

The present study examined post-error neuromuscular adjustments during a TKD-specific kicking task using a within-subject trial-by-trial comparison between Standard Go trials and post-error Go trials. This design allowed the characterization of immediate post-error neuromuscular adaptations within the same athlete across consecutive performance states. The main findings indicate that post-error Go trials were characterized by alterations in the muscle onset latency, particularly in posterior lower-limb muscles associated with propulsion and preparatory force generation during kicking (ST, BF, LG, and SO). Importantly, these adjustments occurred primarily in muscle onset latency, whereas peak EMG amplitude and co-contraction indices remained largely preserved. Furthermore, no significant differences were observed in reaction time between Standard Go trials and post-error Go trials. In the present study, interpretations regarding preparatory motor output are based on the observed differences in muscle onset latency, which was used as an indicator of the temporal organization of muscle activation preceding movement execution [20]. Together, these findings suggest that post-error trials were associated with changes in muscle activation timing rather than with overt behavioral slowing, gross changes in activation magnitude, or generalized modifications in intermuscular coordination. Overall, these findings are consistent with the study’s main hypothesis, namely that post-error adaptation in combat-specific actions would emerge primarily through temporal recalibration of preparatory motor output rather than through behavioral slowing or generalized changes in neuromuscular activation.

A key finding of this study is a systematic temporal shift in muscle activation following error trials, particularly in posterior chain muscles. Given that onset values were referenced to the foot take-off of the kick, with negative values indicating activation prior to foot lift-off, more negative values represent earlier muscle recruitment. In this context, the observed shift toward less negative values in post-error Go trials indicates that muscles were activated closer to the foot take-off. Rather than a simple delay or generalized motor slowing, this pattern suggests a modification in the timing of muscle recruitment output following errors. Importantly, these findings are consistent with the notion introduced in the present study that post-error adaptation may emerge beyond overt behavioral manifestations such as post-error slowing. Although reaction time did not differ significantly between conditions, clear neuromuscular modifications were observed, supporting the idea that the absence of overt behavioral slowing does not necessarily imply the absence of adaptation. Indeed, recent evidence suggests that control-related adjustments may occur even when overt behavioral performance remains relatively stable [30]. Furthermore, timing-related neuromuscular measures may capture subtle adaptations in motor organization that are not necessarily reflected in global behavioral outcomes such as reaction time. Previous EMG research has suggested that temporal and amplitude-related EMG variables represent distinct dimensions of motor control, indicating that changes in the temporal organization of muscle recruitment may emerge independently of overt behavioral performance [31]. Consequently, muscle onset latency may provide complementary information regarding motor organization that is not fully captured by global behavioral outcomes alone [32]. This interpretation is consistent with previous literature indicating that post-error slowing is neither systematically observed nor necessarily associated with adaptive behavioral improvement, suggesting that post-error adaptation may emerge through multiple partially dissociable processes [10,11]. Instead, post-error adjustments may preferentially emerge through subtle recalibration of preparatory motor processes while preserving overt task performance. Such findings reinforce the idea that adaptive motor behavior in combat sports may rely on continuous online recalibration of preparatory motor organization in response to dynamic task demands. Nevertheless, alternative explanations should also be considered. Although the present findings are consistent with post-error motor recalibration, the observed timing shifts may also reflect residual biomechanical or neuromuscular influences from the preceding error trial. Notably, the significant onset-latency changes were observed primarily in posterior lower-limb muscles, which contribute substantially to propulsion during kick execution. Therefore, it is possible that the observed onset shifts partly reflect residual biomechanical states associated with the preceding erroneous movement rather than a generalized post-error adaptation process. Because the present study did not include kinematic measurements capable of characterizing the mechanical state of the limb immediately following the error trial, the relative contribution of adaptive neuromuscular recalibration and residual biomechanical influences cannot be determined. Consequently, the adaptive interpretation proposed here should be considered provisional until confirmed by studies integrating electromyographic and kinematic analyses. More generally, post-error behavioral and motor adjustments may emerge from multiple interacting processes rather than a single adaptive mechanism [10].

In addition, the muscles showing significant onset changes (ST, BF, LG, and SO) are primarily involved in force generation and propulsion during the early phase of the kick. Consequently, the observed temporal recalibration may reflect adaptive regulation of movement preparation in response to previous errors. This interpretation is consistent with evidence indicating that information derived from previous errors can modulate movement preparation and influence the execution of subsequent motor responses [33]. From this perspective, post-error adaptation may involve selective reorganization of the timing of force-generating muscles rather than global suppression of motor output. This interpretation is consistent with the ecological demands of combat sports, in which maintaining rapid response execution may remain essential despite the need for ongoing adaptive regulation following errors [4,15]. Importantly, the preservation of reaction time alongside the observed recalibration of onset further suggests that athletes maintained overt behavioral performance while reorganizing preparatory motor timing at the neuromuscular level.

From a neurophysiological perspective, these findings are consistent with models proposing that preparatory motor regulation involves dynamic modulation of corticospinal excitability during movement preparation [12,17]. One possible explanation is that preparatory inhibitory processes may help reduce background motor noise and optimize signal processing prior to movement initiation [17]. In this framework, error-related processes may transiently influence preparatory motor circuits, potentially contributing to modifications in subsequent motor organization [6]. Importantly, the present findings are consistent with the possibility that such recalibration involves selective modulation of preparatory timing rather than generalized motor suppression. This interpretation is also consistent with previous evidence suggesting that antagonist muscle recruitment and preparatory modulation may represent sensitive markers of inhibitory and adaptive motor processes occurring prior to overt movement execution [13]. Collectively, these findings support the notion that post-error adaptation may be expressed through changes in the temporal organization of preparatory motor output. However, because no direct neural measures were obtained, the underlying mechanisms cannot be determined from the present data, and the proposed neurophysiological interpretations should be considered hypothetical.

Consistent with this interpretation, peak EMG amplitude remained largely unchanged across conditions despite the temporal changes observed in muscle onset latency. This preservation of peak EMG amplitude suggests that post-error adaptation preferentially affected the timing of motor output rather than the global intensity of muscle recruitment. In other words, the neuromuscular system appeared to recalibrate when muscles were recruited rather than how strongly they were activated. This distinction is theoretically important because it suggests that adaptive motor recalibration following errors may emerge through selective temporal adjustments while preserving the overall capacity to generate force. Such findings further support the idea that post-error adaptation may involve fine modulation of preparatory motor regulation rather than generalized reductions in motor output. Accordingly, the present results suggest that post-error adaptation did not involve generalized suppression of the motor system, but rather selective recalibration of preparatory motor timing.

Similarly, co-contraction patterns remained stable across conditions, with no significant differences observed in any muscle pair. The preservation of co-contraction indices suggests that post-error adaptation did not involve global restructuring of intermuscular coordination or joint stabilization strategies. Instead, the stability of coordination patterns alongside the observed onset recalibration supports the interpretation that post-error adaptation was primarily expressed through selective temporal reorganization of preparatory motor processes. Together with the peak EMG findings, these results reinforce the notion that adaptive motor recalibration following errors may preferentially affect preparatory neuromuscular regulation while preserving broader coordination and activation strategies.

Regarding expertise and age, no significant effects were observed across variables, nor were there significant interactions with TrialType. Model comparison further indicated that the inclusion of these predictors did not improve model fit. Although previous work has reported expertise-related differences in neuromuscular organization during kicking execution [21,22,23], the present findings suggest that post-error temporal recalibration mechanisms may operate similarly across expertise levels under the current task demands. Therefore, the exploratory hypothesis that expertise or age would modulate post-error neuromuscular adjustments was not supported. Importantly, these findings do not necessarily imply the absence of expertise-related adaptations in combat sports. Rather, they may indicate that the fundamental mechanisms supporting post-error recalibration of preparatory motor timing are relatively preserved across athletes with different experience levels under the present experimental conditions. Additionally, a substantial proportion of variance was explained by between-subject differences, suggesting the presence of individual neuromuscular strategies. This variability may reflect individual differences in movement organization and adaptive motor regulation during combat-specific actions.

### 4.1. Limitations

First, although the kicking task incorporated ecological motor demands, it cannot fully reproduce the perceptual, tactical, and emotional complexity of real combat situations. In particular, athletes responded to abstract visual stimuli rather than to opponent-derived cues, which may limit the ecological validity and generalizability of the findings to competitive environments. Second, post-error adjustments were examined during a single motor action (roundhouse kick) under relatively stable task constraints. Therefore, the observed neuromuscular adaptations may not necessarily generalize to other TKD techniques, multi-action sequences, or more dynamically interactive situations. Third, although age was included as a covariate in the statistical models, expertise-related effects cannot be completely disentangled from age-related developmental influences due to the age differences between groups. Importantly, age and competitive experience are inherently correlated in TKD athletes, making a complete separation of these factors difficult in cross-sectional studies. Therefore, observed differences between novice and advanced athletes should be interpreted as reflecting the combined influence of competitive experience and age-related developmental factors rather than expertise alone. Fourth, peak EMG amplitude was quantified within a fixed 200 ms window following the detected muscle onset. Although this approach ensured that peak amplitude was evaluated relative to the same activation burst across trials, differences in onset latency between conditions may have shifted the absolute phase of movement represented within the analysis window. Consequently, peak EMG values may not always reflect identical phases of kick execution across conditions, and this should be considered when interpreting the amplitude-related findings. In addition, muscle onset latencies were referenced to foot take-off rather than stimulus presentation, which facilitated the evaluation of movement-related activation patterns but did not allow direct assessment of potential differences in absolute activation timing across conditions. Furthermore, because no kinematic measurements were obtained, it was not possible to determine whether the observed onset-latency shifts reflected adaptive motor adjustments, residual biomechanical influences from the preceding error trial, or a combination of both. Finally, the relatively low frequency of No-Go trials, while necessary to maintain response prepotency (i.e., the tendency to execute the dominant Go response due to its higher probability of occurrence [30]), reduced the number of post-error observations available for analysis. Although linear mixed-effects models are well suited to accommodate unbalanced repeated-measures data, the limited number of post-error observations contributed by some participants may have reduced the precision of individual-level estimates.

### 4.2. Practical Implications

The present findings suggest that error-related motor adjustments in TKD may be expressed through changes in muscle activation timing even when overt behavioral performance remains unchanged. From a practical perspective, coaches and practitioners should not assume that stable reaction-time performance necessarily indicates the absence of post-error motor adjustments. The results also highlight the potential value of wearable EMG systems for identifying subtle changes in movement preparation that may not be evident from behavioral measures alone. In addition, because the observed timing-related changes were primarily detected in posterior lower-limb muscles involved in propulsion and movement preparation, training programs targeting posterior-chain strength, reactive motor control, and rapid inhibition–reinitiation transitions may be useful for enhancing sport-specific motor regulation following errors.

## 5. Conclusions

Post-error Go trials were associated with altered onset-latency patterns in posterior lower-limb muscles, whereas reaction time, peak EMG amplitude, and co-contraction indices remained largely unchanged. These findings suggest that error-related motor adjustments may be expressed through changes in the temporal organization of muscle activation. However, the relative contribution of adaptive motor processes and residual biomechanical influences remains uncertain and should be clarified in future studies integrating electromyographic and kinematic measurements.

## Figures and Tables

**Table 1:** Neuromuscular variables across trial conditions.

Variable	β	95% CI Lower	95% CI Upper	SE	t	*p*	pFDR	Standard Go Trials	Post-Error Go Trials	d
Onset (ms)										
RF	−29.6	−66.7	7.5	18.1	−1.63	0.113	0.170	60.4 ± 88.3	30.8 ± 79.2	−0.31
VL	−19.2	−53.8	15.4	16.9	−1.14	0.265	0.371	125.3 ± 87.4	106.1 ± 66.8	−0.22
VM	−22.0	−65.4	21.5	21.2	−1.04	0.309	0.394	100.0 ± 104.6	78.1 ± 73.7	−0.20
ST	31.2	11.3	51.2	9.8	3.20	0.003	0.006	−250.9 ± 42.1	−219.6 ± 56.0	0.61
BF	31.4	12.8	50.0	9.1	3.47	0.002	0.003	−269.1 ± 30.1	−237.7 ± 43.8	0.66
LG	26.9	6.8	47.0	9.8	2.74	0.011	0.018	−210.3 ± 41.9	−183.4 ± 40.4	0.52
SO	33.7	13.4	53.9	9.9	3.41	0.002	0.004	−232.4 ± 44.0	−198.8 ± 53.4	0.65
TA	14.2	−9.5	37.9	11.6	1.23	0.229	0.332	−212.8 ± 39.1	−198.5 ± 50.1	0.23
Peak EMG (%MVC)										
RF	−0.4	−12.2	11.4	5.8	−0.06	0.949	0.949	66.1 ± 40.3	65.7 ± 23.8	−0.01
VL	6.5	−13.5	26.5	9.8	0.66	0.513	0.598	103.8 ± 41.4	110.3 ± 45.2	0.13
VM	−7.3	−21.5	6.9	6.9	−1.05	0.302	0.394	86.7 ± 37.4	79.4 ± 35.9	−0.20
ST	9.4	1.0	17.8	4.1	2.29	0.030	0.049	40.6 ± 24.7	49.9 ± 33.8	0.43
BF	−7.3	−15.6	0.9	4.0	−1.82	0.079	0.123	67.5 ± 37.0	60.2 ± 47.5	−0.34
LG	−8.1	−26.0	9.9	8.8	−0.92	0.365	0.451	99.3 ± 32.2	91.2 ± 41.1	−0.17
SO	2.3	−21.9	26.6	11.9	0.20	0.845	0.889	129.2 ± 58.8	131.5 ± 60.0	0.04
TA	−0.8	−10.3	8.6	4.6	−0.18	0.856	0.889	34.5 ± 22.6	33.6 ± 17.5	−0.04
Co-contraction index (CCI)										
BF–RF	0.01	−0.010	0.033	0.01	1.08	0.288	0.390	0.17 ± 0.06	0.18 ± 0.08	0.21
VM–BF	0.00	−0.017	0.020	0.01	0.17	0.868	0.889	0.19 ± 0.06	0.19 ± 0.07	0.03
VL–ST	−0.01	−0.025	0.011	0.01	−0.80	0.428	0.514	0.19 ± 0.04	0.18 ± 0.05	−0.15
LG–TA	0.00	−0.015	0.024	0.01	0.50	0.624	0.708	0.25 ± 0.07	0.26 ± 0.08	0.09
SO–TA	0.00	−0.025	0.021	0.01	−0.19	0.852	0.889	0.21 ± 0.08	0.21 ± 0.09	−0.04

Note. β = fixed-effect estimate for TrialType (post-error Go trials vs. Standard Go trials); CI = confidence interval; SE = standard error; pFDR = *p*-value adjusted using the Benjamini–Hochberg false discovery rate procedure; d = Cohen’s d. Onset values are expressed in milliseconds (ms), peak EMG in percentage of maximum voluntary contraction (%MVC), and co-contraction indices are dimensionless. Negative onset values indicate activation occurring prior to the reference event. RF, rectus femoris; VL, vastus lateralis; VM, vastus medialis; ST, semitendinosus; BF, biceps femoris; LG, lateral gastrocnemius; SO, soleus; TA, tibialis anterior.

## Data Availability

The datasets generated and/or analyzed during the current study are available upon reasonable request from the corresponding authors.

## Abbreviations

The following abbreviations are used in this manuscript:

TKD	Taekwondo
PES	Post-error slowing
EMG	Electromyography
MVC	Maximum voluntary contraction
CCI	Co-contraction index
RT	Reaction time
LMM	Linear mixed-effects model
AICc	Akaike Information Criterion corrected
FDR	False discovery rate
